# *Corynebacterium jeikeium* Dormant Cell Formation and Photodynamic Inactivation

**DOI:** 10.3389/fmicb.2020.605899

**Published:** 2020-12-18

**Authors:** Margarita Shleeva, Alexander Savitsky, Arseny Kaprelyants

**Affiliations:** Federal Research Centre “Fundamentals of Biotechnology” of the Russian Academy of Sciences, A.N. Bach Institute of Biochemistry, Moscow, Russia

**Keywords:** *Corynebacterium jeikeium*, resuscitation, porphyrin, photodynamic inactivation, dormant bacteria, resistance

## Abstract

Pathogenic non-spore forming bacteria enter a dormant state under stressful conditions, which likely allows them to acquire resistance to various antibiotics. This work revealed the efficient formation of dormant “non-culturable” (NC) *Corynebacterium jeikeium* cells in stationary phase upon gradual acidification of the growth medium. Such cells were unable to form colonies and existed in a prolonged stationary phase. At an early stage of dormancy (approximately 14 days post-inoculation), dormant cells are able for resuscitation in liquid medium. However, those stored for long time in dormant state needed addition of supernatant taking from active *C. jeikeium* cultures for successful resuscitation. NC cells possessed low RNA synthesis and significant tolerance to antibiotics (rifampicin and vancomycin). They also accumulated free porphyrins, and 5-aminolevulinic acid addition enhanced free porphyrin accumulation which makes them potentially sensitive to photodynamic inactivation (PDI). PDI of dormant bacteria was accomplished by exposing cells to a 565 nm wavelength of light using a SOLIS-4C light-emitting diode for 60 min. This revealed that increased porphyrin concentrations were correlated with elevated PDI sensitivity. Results shown here demonstrate the potential utility of employing PDI to minimize levels of dormant, persistent corynebacteria and the *C. jeikeium* dormancy model developed here may be useful for finding new drugs and techniques for combatting persistent corynebacteria.

## Introduction

Interest in the study of dormant forms of non-sporulating bacteria is caused, on one hand, by the desire to understand long-term bacterial survival mechanisms under conditions that are not conducive to growth that occur in natural ecosystems (El-Registan et al., 2006; Lennon and Jones, 2011). On the other hand, the occurrence of dormancy is associated with pathogen persistence via the formation of antibiotic-resistant, persistent cells (Keren et al., 2004; Zhang, 2004; Lewis, 2007). The problem of antibiotic resistance and the emergence of antimicrobial resistant strains has become especially important in connection with current infectious disease expansion. For example, resistance has been observed in *Staphylococcus aureus*, which is able to form cells with increased antibiotic resistance, which enhances persistence in the host organism and causes chronic or recurrent infections (Chambers and Deleo, 2009). A similar phenomenon has also been described in *Mycobacterium tuberculosis* (Kaprelyants et al., 2018).

The bacterial transition to a dormant state has been documented in experimental studies that described the process in a number of non-sporulating bacteria (Kaprelyants et al., 1993). Further, dormant bacterial cells may develop “non-culturability” (NC) (a term which reflects the inability to form colonies on agar plates), which prevents the cells from being detected *in vivo* using routine plating procedures (Roszak and Colwell, 1987; Cellini et al., 1994; Porter et al., 1995; Kell et al., 1998; Barer and Harwood, 1999; Tholozan et al., 1999; Biketov et al., 2000; Currás et al., 2002; Shleeva et al., 2002; Mukamolova et al., 2003; Wood et al., 2005; Lee et al., 2007). For many non-sporulating gram-negative and gram-positive bacteria, including non-pathogenic relatives of pathogens, morphologically differentiated dormant forms have previously been described (Cellini et al., 1994; Currás et al., 2002; Shleeva et al., 2002, 2004; Lee et al., 2010; Mulyukin et al., 2010). Studying the possible persistence and development of antibiotic resistance of other pathogenic and conditionally pathogenic bacteria, including representatives of the genus *Corynebacterium* is also relevant (Blokpoel et al., 2005; Tauch et al., 2005; Soriano et al., 2009; Olender, 2012).

Many corynebacteria are pathogenic (Bernard, 2012). Corynebacteria of the species *Corynebacterium diphtheriae*, which produce a very strong exotoxin, are the causative agents of one of the most famous human infections, diphtheria. So-called non-diphtheria corynebacteria (Corynebacteria non-diphtheriae) *C. ulcerans* and *C. pseudotuberculosis* cause diphtheria-like diseases that include pseudomembranous pharyngitis, moderate pharyngitis, otitis, lymphadenitis and skin ulcers. *C. minutissimum* is a causative agent of erythrasma and chronic pseudomycosis. *C. amycolatum*, *C. urealyticum*, and *C. striatum* are particularly resistant to penicillins, aminoglycosides and quinolones. *C. jeikeium* strains are causative agents of hospital infections. *C. jeikeium* is responsible for a number of nosocomial infections such as endocarditis, device-connected infection, osteomyelitis (van der Lelie et al., 1995; Mookadam et al., 2006). The bacteria has often found in cancer patients with compromised immune system, inserted in medical instruments, skin lesions, and after antibiotic therapy (Funke et al., 1997). A high mortality rate was documented for *C. jeikeium* sepsis in hematological patients (van der Lelie et al., 1995), and immunocompromised patients carrying prosthetic valves or catheters are particularly susceptible to infection. Further, the curing of *C. jeikeium* frequently limited by developing of multidrug-resistance of the bacteria (Olson et al., 2009; Ifantidou et al., 2010).

Whilst the phenomenon of dormancy in non-sporulating bacteria is extensively studied for many years, corynebacteria dormancy *in vivo* and *in vitro* did not attract much attention. Only one model has been used to assess dormant forms of *C. pseudodiphtheriticum*, which is based on the fivefold limit of the nitrogen source provided in growth media (Mulyukin et al., 2014).

After infecting humans, bacteria are typically captured by macrophages where they are influenced by a number of stresses including low pH, elevated levels of the active forms of oxygen and nitric oxide and the activity of lysosomal hydrolases (Rook et al., 2001; Hacker et al., 2016; BoseDasgupta and Pieters, 2018). However, some bacteria, such as *M. tuberculosis* (Deretic and Fratti, 1999) and *C. ulcerans* (Hacker et al., 2016), can maintain their viability within macrophages despite these harmful factors. Therefore, naturally induced stressful conditions are likely to be factors that are also useful for inducing a dormant state *in vitro*. *M. tuberculosis* (Shleeva et al., 2011) and non-pathogenic *M. smegmatis* (Kudykina et al., 2011) transition to dormant, non-culturable, persistent states in response to the gradual acidification of their environments, therefore, we may suggest that slow decrease in pH levels may result in induction of dormancy in corynebacteria, which, like mycobacteria, belong to the order *Actinomycetales*.

In order to cure chronic infections caused by dormant forms of pathogens, new ways should be established (Kaprelyants et al., 2018). In this regard, application of physical factors seems to be promising in order to destroy metabolically passive dormant bacterial forms. Recently we found that significant concentrations of the intermediates participating in protoporphyrin biosynthesis were present in dormant forms of *M. smegmatis* (Nikitushkin et al., 2016), a fast-growing bacterium which is genetically close to *M. tuberculosis*, These findings suggested that dormant bacteria may be killed by photodynamic inactivation (PDI) when fluorescent porphyrins serve as intracellular photosensitizers. Studies have also demonstrated the photoinactivation of dormant mycobacterial forms *in vitro* in the rapidly growing, tuberculosis-related pathogenic strain, *M. smegmatis* (Shleeva et al., 2019b, 2020).

The goal of this study was to find if *C. jeikeium* are able to form dormant cells as a result of slow decrease of pH level of growth medium in stationary phase. We also tested whether the stimulation of endogenous porphyrin production in dormant corynebacteria enhances their sensitivity to photodynamic inactivation (PDI).

## Materials and Methods

### Organisms and Media

The *C. jeikeium* K411 strain (from State collection of pathogenic microorganisms FSBI Scientific Center for Expert Evaluation of Medicinal Products of the Ministry of Health of the Russian Federation) was grown in TSB broth (Himedia, India) at 37°C for 20–24 h while stirring (200 rpm). A 0.2 mL inoculum was added to 100 mL developed by us 2AS medium (10^5^ cells/mL) with the following composition: 20 g/L glucose; 0.125 g/L MgSO_4_ × 7H_2_O; 1.5 g/L NaCl; 2.5 g/L (NH_4_)_2_SO_4_; 13.6 g/L KH_2_PO_4_; 0.44 g/L histidine (Sigma); 4.0 g/L glutamic acid (Sigma); 8 mL trace element solution; and 0.1% Tween-80. The media was pH adjusted to 6.0 using NaOH. Trace element solution contained 1.0 g/L EDTA, 10.0 g/L MgCl_2_ × 6H_2_O, 0.1 g/L CaCl_2_ × 2H_2_O, 0.04 g/L CoCl_2_ × 6H_2_O, 0.1 g/L MnCl_2_ × 2H_2_O, 0.02 g/L Na_2_MoO_4_ × 2H_2_O, 0.2 g/L ZnSO_4_ × 0.02 g/L 7H_2_O, CuSO_4_ × 5H_2_O and 0.5 g/L FeSO_4_ × 7H_2_O. A final concentration of 0.1% Tween 80 was added. The culture was grown at 37°Ñ in a shaker (200 rpm) for 13–16 days until a pH of approximately 5.5 was established.

### Estimation of Viability

Bacterial suspensions were serially diluted in fresh TSB medium, and three 10 μl samples from each dilution were spotted on TSB (Himedia, India) agar containing 0.1% Tween-80. Plates were incubated at 37°C for 5 days. After incubation, the number of the colony forming units (CFUs) present was counted. The limit of detection was 10 CFU/mL.

The same diluted suspensions (100 μL each) were also used for most probable number (MPN) assays and to evaluate cell resuscitation in 48-well Corning microplates containing appropriate medium (0.9 mL) (see below). Microplates were incubated at 37°C for 14 days without agitation. Wells with visible bacterial growth were considered positive. The number of cells that remained intact after exposure to damage-inducing effects was also determined microscopically by counting propidium iodide (PI)-negative cells in a Helber’s chamber (no less than 10 large fields were counted for each sample).

### Measuring Levels of ^3^H-uracil Incorporation to Determine the Metabolic Activity of Cells

One milliliter samples from cells suspensions were incubated with 1 μl [5,6-^3^H] uracil (10 μCi; 0.2 μmol in 50% ethanol) and incubated for 2 h at 37°C with agitation (45–60 rpm). Cells were then harvested on glass fiber GFC filters (Whatman, United Kingdom) and washed with 3 mL 7% trichloroacetic acid. Next, cells were washed using 3 mL absolute ethanol. Air-dried filters were placed in scintillation liquid and incorporated radioactivity was measured using a scintillation counter (Beckman, United States).

### Spent Medium Preparation

Supernatants (SN) with resuscitating activity were obtained from *C. jeikeium* cultures grown in either TSB or 2AS (initial pH 7.0) media after consecutive sub-culturing. First, a 0.2 mL stock culture (stored at −70°C) was used to inoculate 100 mL culture medium to produce an initial density of approximately 10^3^ cells/mL. The cultures were incubated overnight with agitation (100 rpm). Cells were sub-cultured by transferring a 0.1 mL inoculum to 100 mL fresh medium and cultivating as previously described for 2–30 h. Then cultures were subjected to centrifugation (12,000 × g, 20 min) and sterilization using 0.22 μm filters (Whatman). Fifty milliliters volumes of SN produced in this manner were frozen and stored at −70°C. *C. jeikeium* growth stimulating activity of SN was assessed after SN was added to freshly inoculated TSB or 2AS media at a 1:1 vol/vol ratio. Stored SNs were used immediately after thawing and were not refrozen and reused.

### Resuscitation of “Non-culturable” *C. jeikeium* Cells

Non-culturable cells were separated from spent medium (centrifugation for 20 min at 5, 000 × g), that was serially diluted and used to inoculate either TSB or 2AS medium. An equal volume of either SN was prepared as indicated above, or appropriate uninoculated medium (control), and numbers of viable resuscitated cells were determined using the MPN assay (de Man, 1974).

### Sensitivity to Antibiotics and Heat Treatment

Four milliliters of an early stationary phase (ESP) culture that was grown in TSB medium (pH 7.0) for 1 day or 4 mL of a 25 days old culture incubated at reduced pH were treated with 0–10 μg vancomycin/mL or 0–100 μg rifampin/mL and incubated at 37°C or room temperature for 24 h without agitation. The number of resistant cells was determined using the MPN assay in the presence of TSB culture SN (5 h, see above). One milliliter samples of ESP cultures or 25 days long incubated suspensions containing dormant cells were heated to 60, 65, 70, 75, or 80°C for 10 min, and the number of survivors within samples were determined by evaluating the growth of bacteria in presence of SN (MPN assay).

### Pigment Extraction From the Cells

Pigment was extracted from the biomass in accordance with the method described by Bligh and Dyer (Bligh and Dyer, 1959). First, 1 mL chloroform and 2 mL methanol were added to the wet biomass of cells (0.8 g). Cells were agitated for 12 h in the extraction mixture and subsequently centrifuged (2,000 × g), followed by the addition of 1 mL water and 1 mL chloroform (to separate phases). The chloroform layer was washed three times with 0.1 M NaCl. The bacterial pellet was re-suspended in ice-cold 100 mM HEPES (4-(2-hydroxyethyl)-1-piperazineethanesulfonic acid) buffer (pH 8.0) containing 2% Triton × 100 (Sigma, United States) then lysed by using zirconium beads on a bead beater homogenizer (MP Biomedicals FastPrep-24) for 1 min, five times and stayed in dark place at room temperature for 5 h. The bacterial lysate was centrifuged at 13,000 rpm for 15 min at 4°C.

### Absorption and Fluorescence Spectra

Absorption spectra were recorded on a Cary 300 Bio Spectrophotometer (Varian, Inc., United States). Fluorescence measurements were carried out with a Varian Cary Eclipse fluorimeter (Varian, Inc., United States).

### PDI

Either dormant or active cell suspensions were used for light-inactivation experiments. *C. jeikeium* suspensions with OD values equal to 0.1, which corresponded to approximately 10^7^ bacteria/mL, were used. Bacterial suspensions (100 μL) were added to the wells of a Nunc 96-well plate (ThermoFisher Scientific, United States). Samples were illuminated with a SOLIS-4C light-emitting diode at 565 nm, 3.2 W using bandpass filter MF565-24 (Thorlabs, United States). The light beam was collimated to a diameter of 5 mm, which corresponded to the diameter of the wells of the 96-well plate, and samples were illuminated for 60 min. The power density of the light was 145 mW per well (513 mW/cm^2^) as determined using a 2,936-c power meter (Newport, United States). Temperature was controlled an accuracy of ± 0.2°C by placing an 80BK type-K multimeter thermocouple (Fluke, Germany) directly in the microcell before and after lighting in the presence and absence of bacterial suspension. Temperatures were below 40°C in the wells of all samples. After the illumination of samples, numbers of viable bacteria were estimated using an MPN assay in presence of 5-h-old SN obtained from TSB medium (see above).

### Microscopy

Cell suspensions were examined under a microscope (Eclipse E4000, Nikon, Japan) in phase-contrast and epifluorescence modes after staining with propidium iodide (PI) (3 mM) to detect injured cells, ethidium bromide (5 mM) to detect DNA-containing cells, or Nile red (4 mg/mL) to detect lipid inclusions. The excitation value was at 510 nm and emission was at > 560 nm.

### Statistical Analysis

Statistical processing was carried out using the analysis of the standard deviation or relative error within the data group. MPN values were determined using de Man’s tables calculated on the base of Poisson distribution (de Man, 1974). For the MPN assay (95%) confidence limits were calculated. The MPN values were considered statistically different if low and high confidence limits were not overlapped.

## Results

### Acidification of the Medium Induces Dormancy in *in vitro* Culture of *C. jeikeium*

**FIGURE 1:**
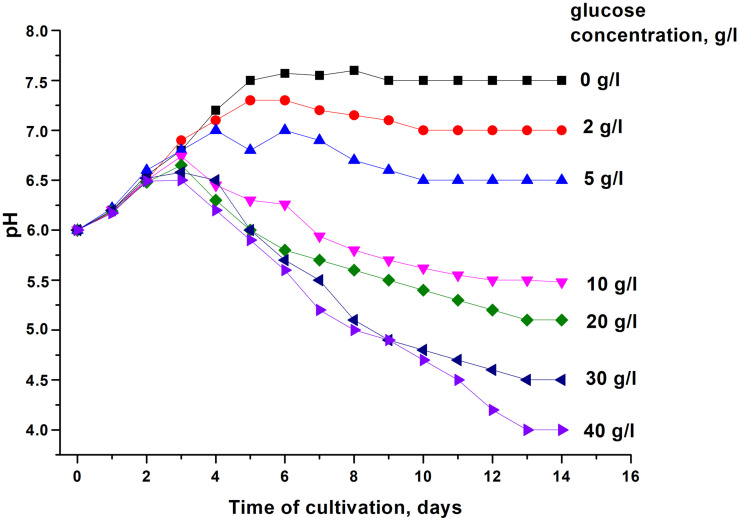
Changes in the pH of media with different concentrations of glucose upon cultivation of *Corynebacterium jeikeium. C. jeikeium* strain K411 was initially grown for 1 day in TSB medium supplemented with Tween-80. The culture grown in this medium served as an inoculum and was added to 2AS medium (initial concentration 10^5^ cells/mL; initial pH = 6.0) and grown while agitating at 37°C for 14 days. Samples of *C. jeikeium* cultures were taken periodically from the same flask and passed through a 0.2 μm filter (Whatman) to obtain supernatants for pH measurements. Relative error of pH measurements was about 3%. The experiment was repeated three times, and a typical result is shown.

**FIGURE 2:**
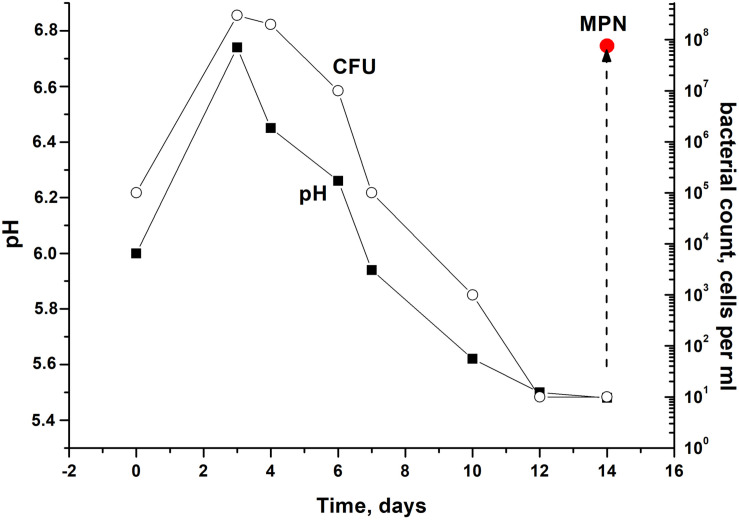
Culturability of *Corynebacterium jeikeium* under medium levels of acidification in a prolonged stationary phase. *C. jeikeium* strain K411 was initially grown for 1 day in TSB medium supplemented with Tween-80. The culture grown in this medium served as an inoculum and was added to 2AS medium. Each inoculum (10^5^ cells per ml) was added to flask with 2AS medium (initial pH6.0, 10 g/L glucose) and grown while agitating at 37°C for 14 days. Samples of cultures were taken from flasks to determine pH, CFU, MPN and the CPM. MPN was estimated in the presence of supernatant taken from an actively growing *C. jeikeium* culture (see section “Materials and Methods”). Data shown are results of a typical experiment (five biological replicates). The relative error for CFU ranged between 10 and 30%. The relative error of pH values was about 3%. Bars represent (95%) confidence limits for the MPN assay.

In order to modulate changes in pH values during the stationary phase of *C. jeikeium* culture we used developed by us a synthetic medium (2AS) supplemented with different glucose concentrations (0–40 g/L) (Figure 1). After a phase of modest alkalization (to approximately pH 6.8), the gradual acidification of the culture to distinct final pH values were observed for glucose concentrations that ranged from 10 to 40 g/L. More significant pH decreases were observed in stationary phase cultures in 2AS medium containing 30–40 g/L glucose (Figure 1). In cultures grown with 10–20 g/L glucose, stationary phase of growth was established within approximately 3 days, and further incubation resulted in the gradual decrease of CFU values, starting about 4 days post-inoculation (Figure 2). A minimum CFU was found between 12 and 14 days after inoculation, which indicated that the cultures had transitioned to “non-culturability.”

After the exponential growth, total cell counts (according to microscopy) and OD measurements were maintained at the constant level of 2.3 ± 0.8 × 10^9^ cells per mL for total count and 4.8 ± 0.2 for OD_600_. The exact time point at which culturability (according to CFU counts) demonstrated minimum values varied in different experiments. However, a correlation between culturability and culture acidification was evident (Figure 2).

**FIGURE 3:**
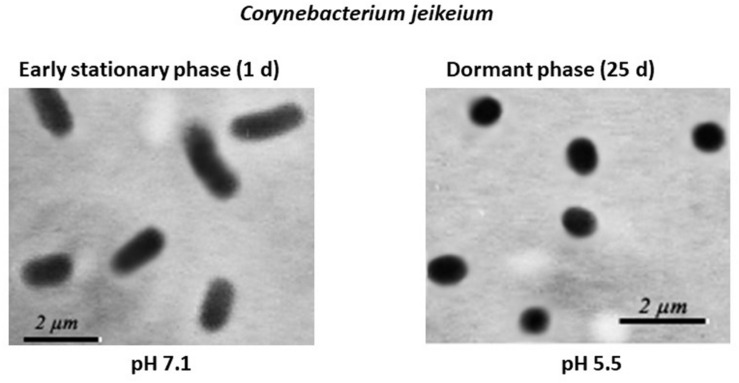
Morphological changes in post-stationary phase *Corynebacterium jeikeium* cells throughout the gradual acidification of the growth medium. *C. jeikeium* strain K411 was initially grown for 1 day in TSB medium supplemented with Tween-80. The culture grown in this medium served as an inoculum and was added to 2AS medium. Each inoculum (10^5^ cells/mL) was added to 2AS medium (initial pH6.0, 10 g/L glucose) and grown while agitating at 37°C for 14 days. Active bacteria were grown using TSB medium for 20 h while agitating at 37°C. Culture samples were taken from flasks and examined under a phase-contrast microscope (1,000x magnification).

Microscopic examination of non-culturable *C. jeikeium* grown at 10–20 g/L glucose and stabilized at pH 5.5 by adding 50 mM MES (2-(N-morpholino)ethanesulfonic acid) buffer revealed that the majority of the cells displayed coccoid forms that were approximately 1 ± 0.5 μm in length. This was in contrast with characteristics of exponentially growing cells, which typically possess elongated ovoid shapes, are 2 μm in length, and have an average diameter of 1 μm (Figure 3). A microscopic assessment of dormant *C. jeikeium* cultures revealed that the majority of bacterial cells remained intact when incubated 30 days in liquid medium at room temperature, according to a PI test. When the cellular permeability barrier is destroyed, PI penetrates through cell wall and binds with DNA producing a fluorescent complex. According to this test 28 ± 5% of PI-positively stained cells were found in stabilized dormant population vs. 5–10% stained cells found in active cultures (Supplementary Figure 1). However, if the final pH value of the external environment was below 4.8, a significant number of dead bacteria appeared in the culture (Figure 1 and Table 1).

**TABLE 1:** Medium pH, metabolic activity and percentage of dead cells in 14 days old C *jeikeium* cultures growns in 2AS medium supplemented by different glucose.

**Glucose concentration g/l**	**0**	**2**	**5**	**10**	**20**	**30**	**40**
**Parameter**							
Medium pH	7.5	7.0	6.5	5.48	5.1	4.5	4.0
% dead bacterials cells (PI+)	5 ± 3	4 ± 2	7 ± 4	10 ± 2	15 ± 5	40 ± 8	90 ± 5
Incorporation of H^3^-uracil CPM/mg wet cell weight	7,512 ± 354	7,244 ± 265	5,325 ± 254	62.7 ± 22	50.5 ± 18	20.2 ± 5	18.4 ± 4.5

*The mean ± SD is shown.*

### Dormant *C. jeikeium* Cells Are Characterized by Low Metabolic Activity

A metabolic activity assay was performed that estimated metabolic activity by evaluating levels of ^3^H-uracil incorporation. Cultures in which acidification was observed possessed significantly reduced levels of ^3^H-uracil incorporation relative to cultures in which pH values were maintained at neutral levels (Table 1). Under conditions where the maximum percentage of dormant intact cells was produced (10–20 g/l glucose, final pH 5.0–5.5), RNA synthesis activity was less than 1% of that for cells grown under neutral pH (Table 1).

### Resuscitation of Dormant *C. jeikeium* Cells

**FIGURE 4:**
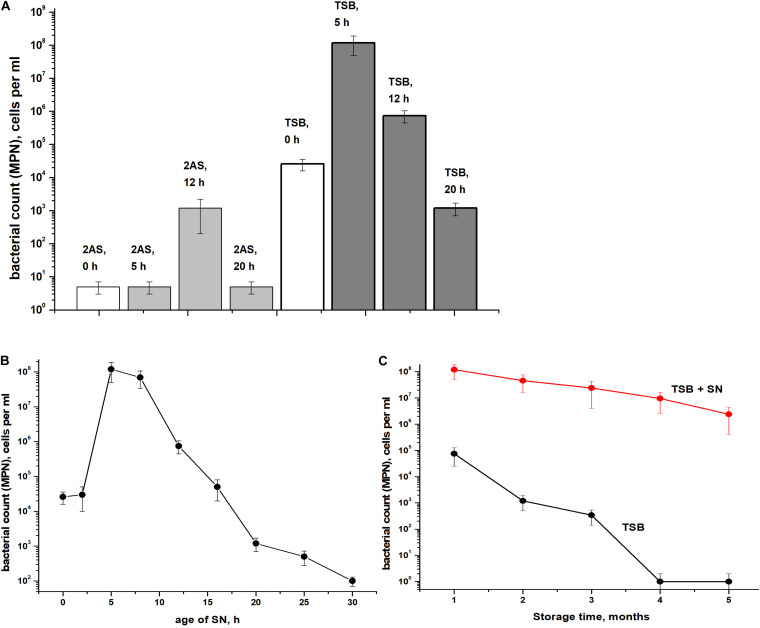
Resuscitation of dormant *Corynebacterium jeikeium* cells. **(A)** A *C. jeikeium* culture was harvested at the period of minimum culturability (14 days, Figure 2). MPN assays were performed in TSB or 2AS medium supplemented with 0.1% Tween-80 that possessed and lacked SN taken from cultures of *C. jeikeium* (5–20 h) grown in TSB or 2AS medium. The results of 8 different experiments are summarized and SD values are given. **(B)** Resuscitation was also measured on TSB media using SNs of cultures of different ages. Results of a representative experiment are shown. **(C)** Resuscitation of dormant *C. jeikeium* cultures with different periods of storage at acid conditions as describe in M&M. Cell viability was estimated using an MPN assay in the presence or without of TSB SN. Bars represent (95%) confidence limits for the MPN assay. The experiments were repeated two times, and a representative result is shown.

The presence of ca. 90% of PI-negative coccoid cells in *C. jeikeium* cultures in the post-stationary phase of growth in presence of 10 g/l glucose (14 days) (Table 1) and ca. 70% for 30 days old dormant cultures (Supplementary Figure 1) assumed that these morphologically changed cells (Figure 3) had kept their integrity and therefore potential viability, despite a sharp decrease in CFU number (Figure 2). Their potential viability was confirmed in resuscitation experiment via a MPN assay (Figure 2). After *C. jeikeium* cultures containing coccoid cells were incubated 10–14 days after inoculation when CFU number had decreased up to zero, the number of viable cells estimated by MPN assay in liquid TSB medium reached 10^5^ cells/mL (Figure 4A). Whilst the cultures further incubated for up to 5 months, the number of cells is able for resuscitation decreased up to undetectable level (Figure 4C). However, these cells could be resuscitated when supernatant obtained from replicating *C. jeikeium* was added to the resuscitation medium. Supernatant with maximum resuscitation activity was obtained from a growing in TSB medium for 5–12 h *C. jeikeium* cultures (Figures 4A,B). Samples stored as long as 5 months revealed concentration of potentially viable cells between 10^7^ and 10^8^ cells/mL which could be resuscitated upon addition of SN (Figure 4C).

As revealed by microscopy, resuscitation of coccoid *C. jeikeium* cells resulted in their conversion to normal bacilli. According to 16S RNA sequences coccoid *C. jeikeium* cells after resuscitation shown 100% identity to *C. jeikeium*.

### Dormant *C. jeikeium* Cells Are Resistant to Heat and Antibiotics

**FIGURE 5:**
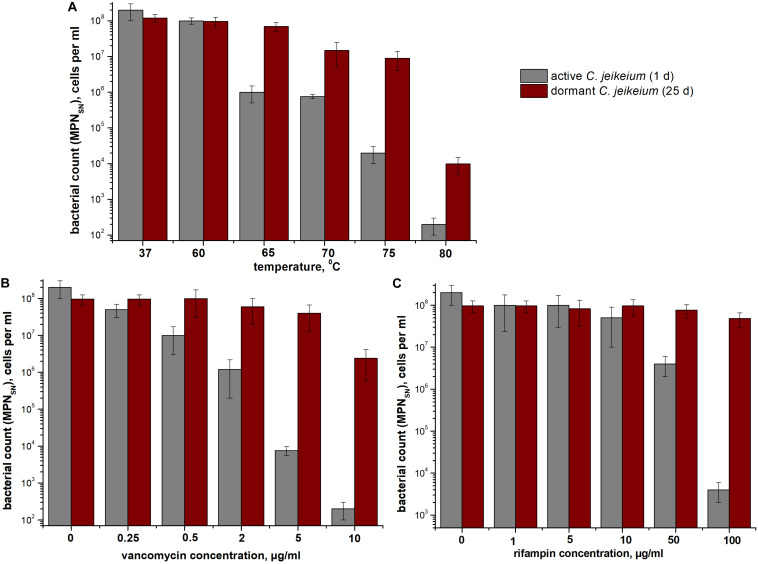
Bacterial resistance to **(A)** high temperatures and **(B,C)** antibiotics. The sensitivity of *Corynebacterium jeikeium* cells to heating or antibiotic treatment is shown. Samples were taken from 25 days cultures that contained coccoid cells. For comparison, *C. jeikeium* cells were cultivated in TSB medium for 20 h (active cultures). Both types of cells were heated to 60–80°C for 10 min **(A)**, treated with 0–10 μg vancomycin/mL **(B)**, or treated with 0–100 μg rifampin **(C)** and incubated at 37°C or room temperature for 24 h without agitation. Cell viability was estimated using an MPN assay in the presence of SN taken from culture grown in TSB medium for 5 h (for details see section “Materials and Methods”). Active cells are shown using gray columns and dormant cells are indicated using red columns. Bars represent (95%) confidence limits for the MPN assay. The experiments were repeated three times, and a representative result is shown.

According to MPN assays, coccoid cells from 25-days-old cultures were more resistant to heat treatment than exponentially growing cells (Figure 5A). As opposed to early stationary-phase cells, coccoid *C. jeikeium* cells were much more resistant to treatment with vancomycin (0.5–10 μg/mL) (Figure 5B) or rifampicin (50–100 μg/mL) (Figure 5C) for 1 day than early stationary phase cells, as revealed by MPN viability tests in presence of SN.

### Accumulation of Porphyrins by Dormant *C. jeikeium* Cells

**FIGURE 6:**
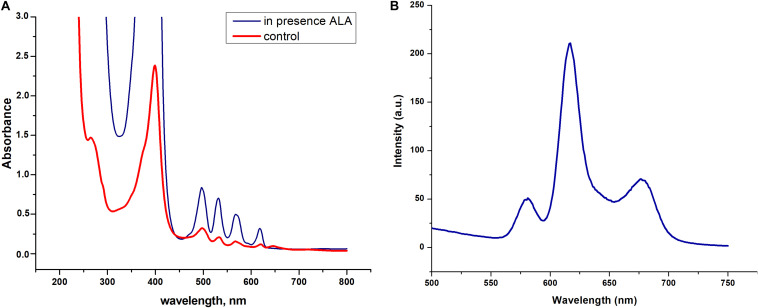
Spectral properties dormant *Corynebacterium jeikeium* pigments. **(A)** Absorption spectra of a Triton X100 *C. jeikeium* extracts obtained from 0.8 g (wet weight) of cells for both cultures. The blue line indicates absorption spectrum of the extract from dormant bacteria grown in presence of 5-aminolevulinic acid. The red line indicates absorption spectrum of the Triton x 100 cellular extract from dormant bacteria grown without ALA (for details see section “Materials and Methods”). **(B)** Typical fluorescence emission spectra of extracted pigments (λ excitation, 400 nm).

We found that dormant *C. jeikeium* cells accumulate a pigment characterized by the typical absorption spectra for porphyrins (Soret band at 400 nm and by emission maxima of fluorescence in the range of 620–670 nm) (Figures 6A,B). The intracellular pigment concentration had the potential be increased if a precursor of porphyrin synthesis, 5-aminolevulinic acid (ALA) was added to the growth medium in accordance with spectral values produced by chloroform–methanol or Triton X100 extracts of dormant bacteria (Figure 6A).

### Photoinactivation of Dormant *C. jeikeium* Cells

**FIGURE 7:**
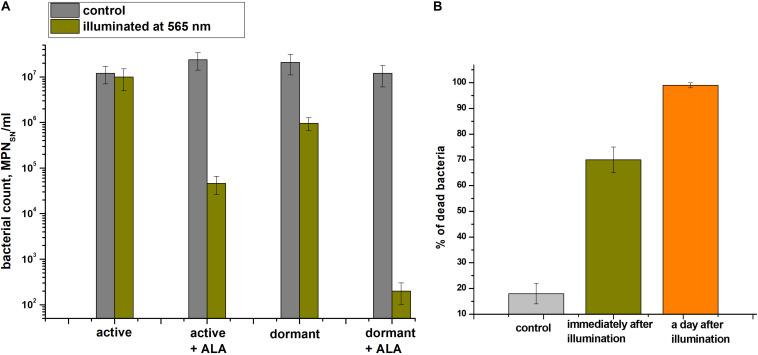
Photodynamic inactivation of dormant *Corynebacterium jeikeium* at 565 nm. Dormant and active cells in the presence or absence of ALA were obtained and subjected to PDI as described in section “Materials and Methods.” **(A)** After 60 min of exposure under static conditions, numbers of viable bacteria were estimated via an MPN assay using 5-h-old SN of TSB cultures. Bars represent (95%) confidence limits for the MPN assay. **(B)** Percentage of damaged dormant *C. jeikeium* cells estimated by PI staining microscopically immediately after illumination and 24 h later is shown. Bars represent ± SD. Controls are unilluminated cells. The experiments were repeated three times, and a representative result is shown.

The effect providing 565 nm wavelength light (which coincides with the absorption of porphyrins in the visible region) on the viability of active and dormant corynebacterial forms grown in the presence and absence of 1.0 mM ALA was studied (Figure 7A). The greatest decrease in the corynebacterial survival rate was observed when light was applied to dormant *C. jeikeium* formed in the presence of ALA in which a 4.5-log10 decrease in survival after 60 min of illumination was observed (Figure 7A). In case of cells taken from the early stationary growth phase (20 h), illumination did not reduce survival (MPN), but in presence of ALA photosensitivity increased (a 3-log10 decreased in viability was observed after illumination) (Figure 7A).

Microscopy was used to demonstrate that the illumination immediately resulted in the appearance of injured cells (Figure 7B). After 60 minutes of light exposure, about 68 ± 5% of the dormant *C. jeikeium* cells formed in the medium contained ALA were damaged. In contrast, only 18 ± 4% of unilluminated cells were damaged. 24 h later, this proportion of damaged cells in the population was greater than 99% (Figure 7B).

## Discussion

In this study, we ascribe conditions needed for corynebacterial cells to transition to a state of dormancy, which was accompanied by: (1) the formation of stress-resistant forms intended for long-term survival that have been identified in many spore-forming and non-spore-forming bacteria (Zhang, 2004; Mulyukin et al., 2010, 2014; Lennon and Jones, 2011) and (2) the acquisition of an NC state during the prolonged incubation of post-stationary cultures, which was previously described for *Micrococcus luteus* (Kaprelyants and Kell, 1993), *Rhodococcus rhodochrous* (Shleeva et al., 2002) and mycobacteria (*M. tuberculosis* and *M. smegmatis*) (Shleeva et al., 2003, 2004, 2011, 2015; Kudykina et al., 2011). The dormant forms of mycobacteria, which form when the external medium undergoes gradual acidification, can be distinguished from active bacteria based on their distinct proteomic (Trutneva et al., 2018, 2020) and metabolomic (Nikitushkin et al., 2020) profiles.

*C. jeikeium* cells formed in post-stationary cultures should be classified as dormant bacterial forms based on the following features: (i) the retention of viability throughout an extended incubation period (up to 5 months) (Figure 4), (ii) their low level of metabolism (Table 1), (iii) their enhanced resistance to deleterious factors such as high temperatures and antibiotic treatment (Figure 5), (iv) the acquisition of an NC state. In the NC state, cells maintain viability (the potential for proliferative activity), but could not produce colonies on agar media and, therefore, are not detected using standard tests (Colwell and Grimes, 2000; Oliver, 2010). The determination of whether non-sporulating *C. jeikeium* have the ability to transition to a long-lived dormant form of the bacteria has the potential to broaden our understanding of the mechanisms by which bacteria survive in natural systems when they encounter conditions not conducive for growth.

The NC state can be reversed by applying a resuscitation procedure in MPN assay either in fresh medium or in the presence of SN. MPN assay was used to evaluate a number of potentially viable cells in cultures that were diluted to disappearance in liquid medium. At the same time this approach permitted the resuscitation of cells that were deprived of the ability to produce colonies on agar plates. This procedure was previously developed to resuscitate mycobacteria by cultivating “non-culturable” cells in liquid medium (Shleeva et al., 2011). The increased necessity of SN-based resuscitation as *C. jeikeium* cultures age could be due to the existence of cells in different physiological state: those that are simply unable to form colonies on plates and those that are at the depth of dormancy. Previously, we have found that a secreted protein Rpf (resuscitation promoting factor) provided culture SN activity for *Micrococcus luteus* (Mukamolova et al., 1998). The protein stimulates the resuscitation of non-culturable cells and shortens the lag phase of active *M. luteus* cultures inoculated with a low dose of bacteria. Rpf family is comprised of peptidoglycan hydrolases (Kana and Mizrahi, 2010) and the products of their enzymatic activity stimulate the reactivation of dormant mycobacteria (Nikitushkin et al., 2013, 2015). The expression of the *rpfA* gene in *M. tuberculosis* is under control of the transcriptional regulator Rv3676 and a cAMP receptor protein (CRP) (Rickman et al., 2005). Presumably, a protein similar to the Rpf protein in *M. luteus* is the active factor present within the cell free *C. jeikeium* culture liquid. Indeed, the *C. jeikeium* genome contains three genes encoding proteins similar to Rpf (*JK_RS02150/jk0416*, RpfA; *JK_RS07760/jk1512*, RpfB; *JK_RS00265/jk0051*, RpfC), which may be released into the environment and function to resuscitate NC *C. jeikeium* cells.

However, other reactivation stimulants, such as phospholipids (Zhang et al., 2001), free unsaturated fatty acids (Nazarova et al., 2011), cAMP (Shleeva et al., 2013), muropeptides (Nikitushkin et al., 2013), may also be present within the supernatant of actively growing bacterial cultures. It was previously shown that unsaturated fatty acid-dependent adenylate cyclase Rv2212 and a cAMP-dependent transcription factor of the Crp family Rv3676 participate in the reactivation of dormant forms of *M. tuberculosis* (Shleeva et al., 2017, 2019a). There is also a link between the Crp factor Rv3676 and RpfA protein synthesis (Bai et al., 2005). The *C. jeikeium* genome contains a gene encoding a cAMP-dependent transcriptional regulator of Crp family, glxR (*JK_RS10065/jk1972*), which may have a function that is similar to Rv3676.

It is clear that dormant cells could be of significant importance for medicine and microbiology due to their ability to form normal, viable organisms after resuscitation (Kaprelyants et al., 1993; Barer, 1997; Barer et al., 1998; Kell et al., 1998; Barer and Harwood, 1999; Mukamolova et al., 2003). For example, diphtheria bacteriocarrier phenomenon is known, and is considered one of the mechanisms by which a pathogen can persist within the host organism (Deviatova, 1956; DeWinter et al., 2005; Kostyukova and Bechalo, 2018).

*C. jeikeium* cells are resistant to a number of many beta-lactam antibiotics, doxycycline and ciprofloxacin, but they are sensitive to vancomycin and rifampicin (Soriano et al., 1995). However, the dormant forms of *C. jeikeium* used in the study that were produced as a result of the acidification of the environment proved to be resistant to both rifampicin and vancomycin (Figures 4B,C). Researchers previously demonstrated that, in some cases, vancomycin may be ineffective in the treatment of *C. jeikeium* bacteremia and endocarditis and disease recurrence occurred despite providing an extended course of antimicrobial therapy (Vanbosterhaut et al., 1989; Clarke et al., 2019). Wang et al. (2001) “retrospectively reviewed 53 cases of *C. jeikeium* bacteremia in bone marrow transplant recipients who had a Hickman catheter without signs of local catheter site infection. The results showed that salvage of catheter with vancomycin therapy is successful in most patients (93%)” (Bookani et al., 2018). However, 7% of patients that underwent catheter salvage were affected by recurrent bacteremia. Whilst the genetic characterization of causative pathogen was not performed in the study, the emergence of such resistance of corynebacteria to the antibiotic could be a consequence of the transition of bacteria to a dormant state, which protects them from being killed by antibiotic treatment. The fact that *C. jeikeium* was resistant to both vancomycin and rifampicin allows us to suggest a model of *C. jeikeium* dormancy that can be used to screen for new drugs that possess activity against chronic *C. jeikeium* bacteremia.

Evidently, dormant bacteria with little metabolic activity are capable of avoiding effects of antibiotics even though they remain suitable targets. It can be assumed that the effect of complete elimination of dormant, “non-culturable” bacteria can be accomplished by factors which produce indirect, harmful effects on bacteria (Kaprelyants et al., 2018). In this regard, we employed a previously discovered phenomenon of the accumulation of free and methylated porphyrins in dormant mycobacteria (Nikitushkin et al., 2016). This finding made it possible to induce the PDI of mycobacteria (Shleeva et al., 2019b, 2020). In the present study we found that dormant *C. jekeium* cells accumulate a pigment which according to the spectral properties (Figures 6A,B) evidently belong to a class of porphyrins (Gouterman, 1961).

Of the currently available photosensitizers (PSs) available, porphyrin and its analogs have the following advantages: (1) many are effective producers of singlet oxygen; (2) they usually do not generate singlet oxygen in the absence of light; (3) they absorb in the red region of the optical spectrum; and (4) they are relatively stable. As an alternative to antibiotic treatment for killing dormant *C. jeikeium*, PDI was a promising approach that was used for killing dormant bacteria (Figure 7). In a previous study, an elevated accumulation of coproporphyrin III was observed in *C. diphtheriae* culture filtrates (Gray, 1948). The overproduction of porphyrins was also observed in 15 days old *C. acnes* cultures (Cornelius and Ludwig, 1967). However, the link between porphyrin accumulation and *C. jeikeium* dormancy was not established previously.

The functional role for the porphyrins that accumulate in dormant bacteria is not clear. Likely that newly found, hydrophobic porphyrins may stabilize and protect dormant cells against unfavorable conditions or destructive factors (Patel and Day, 1999; Castello et al., 2008; Antonova et al., 2010). In addition, stored porphyrins may be utilized as metabolites in biochemical pathways during dormant bacterial cell reactivation.

Bacterial killing in association with PDI seems to be due to the production of reactive oxygen compounds, which participate in the oxidation of such important molecules as enzymes, proteins, lipids and nucleic acids, are lethal to the bacterial cell (Demidova and Hamblin, 2004; Jori et al., 2006). PDI is known as a robust approach for killing bacterial cell including multi-resistant bacteria (Grinholc et al., 2008; Cassidy et al., 2010). PDI was found to be efficient for eliminating of a number of bacteria (Plavskii et al., 2018), including virulent *E.coli* O157H7, *Listeria monocytogenes* (Romanova et al., 2003), *Neisseria gonorrhoeae* (Wang et al., 2019) and *Legionella rubrilucens* (Schmid et al., 2019). Commonly, exogenously added photosensitizers, in particular porphyrins, were applied in PDI experiments (Liu et al., 2015; Guterres et al., 2020; Rossi et al., 2020). For example, cyclodextrin with bound porphyrins has been applied for the treatment of *Staphylococcus aureus* (Hanakova et al., 2014). Endogenous porphyrins were utilized for the killing of pathogenic periodontal bacteria (Cieplik et al., 2014; Hope et al., 2016), skin pathogens *St. aureus* (Ashkenazi et al., 2003; Lipovsky et al., 2009), *Staphylococcus carnosus* (Hoenes et al., 2020), *Hemophilis parainfluenzae* (van der Meulen et al., 1997) and *Helicobacter pylori* (Hamblin et al., 2005; Morici et al., 2020).

Apart of photosensitizer, application of 5-aminolevulinic acid (ALA) was employed as another approach. ALA being photodynamically inactive stimulates biosynthesis of endogenous PSs in cells that enhances harmful effect upon illumination (Harris and Pierpoint, 2008; Bohm et al., 2020). In bacteria, close to eukaryotic cells, the biosynthesis of porphyrins from ALA took place, and ALA administrated to the cell culture enhances porphyrin accumulation in the cell (Nitzan et al., 2004).

Application of ALA-PDI in clinic revealed positive results notably for the skin diseases such as acne, chronic folliculitis, rosacea, lichen sclerosis and skin lesions caused by *Mycobacterium marinum*. According to the clinical trials, the usage of ALA-PDI look promising for the treatment of ulcers caused by *Helicobacter pylori* (Wilder-Smith et al., 2002; Nitzan et al., 2004). However, the application of PDI for eliminating both active and dormant corynebacteria was demonstrated for the first time in the present study. This work suggested a new approach that has the potential to be used to eradicate dormant corynebacteria in clinical settings.

## Conclusion

Under gradual medium acidification, *C. jeikeium* cells transit to dormant “non-culturable” state with the retention of viability, low level of metabolism, enhanced resistance to deleterious factors, porphyrins overproduction. This model of *C. jeikeium* dormancy can be used to screen for new drugs that possess activity against persisting *C. jeikeium*. Resistant to antibiotics dormant *C. jeikeium* cells can be eliminated by application of photodynamic inactivation due to the endogenous porphyrin accumulation. The stimulation of porphyrin production by ALA in dormant and active corynebacteria enhances their sensitivity to photodynamic inactivation.

## Data Availability Statement

The original contributions presented in the study are included in the article/Supplementary Material, further inquiries can be directed to the corresponding author/s.

## Author Contributions

AK and MS conceived, designed the experiments, wrote the manuscript, and revised the manuscript. MS and AS performed the experiments. AK, AS, and MS analyzed the data. MS prepared figures and graphs. All the authors read and approved the final manuscript.

## Conflict of Interest

The authors declare that the research was conducted in the absence of any commercial or financial relationships that could be construed as a potential conflict of interest.

## Supplementary Material

Supplementary Figure 1 Percentage of damaged *C. jeikeium* cells. Dormant and active cells were obtained as described in section “Materials and Methods.” Proportion of dead cells was estimated microscopically by counting propidium iodide (PI)-negative cells in a Helber’s chamber. Bars represent ± SD. The experiments were repeated two times, and a representative result is shown.
