# Supplementary figures and images for: Corynebacterium jeikeium Dormant Cell Formation and Photodynamic Inactivation
Source: Front Microbiol. 2020 Dec 18;11:605899. doi: 10.3389/fmicb.2020.605899 (PMC7775403; doi:10.3389/fmicb.2020.605899)

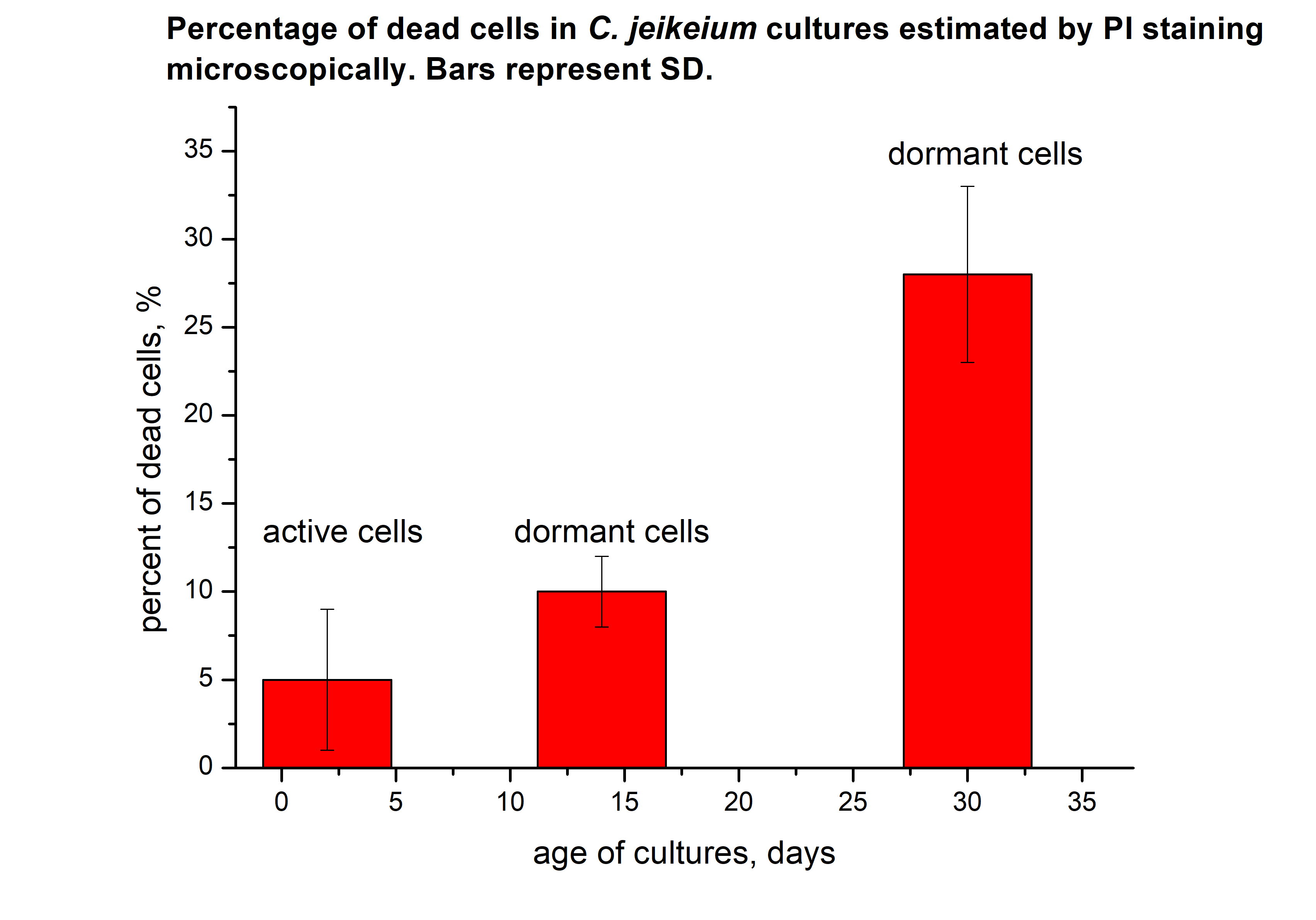

Supplement: Supplementary Figure 1 — Percentage of damaged C. jeikeium cells. Dormant and active cells were obtained as described in section “Materials and Methods.” Proportion of dead cells was estimated microscopically by counting propidium iodide (PI)-negative cells in a Helber’s chamber. Bars represent ± SD. The experiments were repeated two times, and a representative result is shown. [file Image_1.JPEG]
